# Optimizing dosimetric leaf gap parameters for radiation therapy: A comparative study using three diode‐array verification tools and an ionization chamber

**DOI:** 10.1002/acm2.70280

**Published:** 2025-10-28

**Authors:** Yoshitsugu Matsumoto, Ryosuke Sato, Natsumi Futakami, Tsuyoshi Fukuzawa, Ryuta Nagao, Toshihisa Kuroki, Tatsuya Mikami, Yoji Nakano, Yuri Toyoda, Koichi Fukumoto, Akitomo Sugawara

**Affiliations:** ^1^ Department of Radiation Oncology Tokai University School of Medicine Isehara Kanagawa Japan; ^2^ Department of Radiology Tokai University Hospital Isehara Kanagawa Japan; ^3^ Department of Radiation Oncology Tokai University Hachioji Hospital Hachioji Tokyo Japan

**Keywords:** ArcCHECK, Delta4, dose difference, dosimetric leaf gap, gamma index, ionization chamber, SRS MapCHECK

## Abstract

**Background:**

The dosimetric leaf gap (DLG) value is a key parameter that affects dose calculation accuracy in radiotherapy when using the Eclipse treatment planning system. Diode array dosimeters are used to determine the optimal DLG value because they are convenient and fast, providing instant readout of planar dose distributions rather than a point dose. However, variations in gamma pass rates (GPRs) across devices, even for the same radiotherapy treatment plan, may lead to different DLG determinations.

**Purpose:**

To compare the optimal DLG values determined using three ‐diode array dosimeters with those obtained using an ionization chamber (IC) and to evaluate whether diode array dosimeters can reliably reproduce IC‐based DLG values.

**Methods:**

Optimal DLG values were determined for 6 MV, 10 MV, and flattening filter‐free (FFF) beams using ArcCHECK, Delta4, and SRS MapCHECK. The results were compared with the IC‐based values across all energies. Radiochromic films validated the IC‐based DLG values. Twenty volumetric modulated arc therapy plans were recalculated using ten different DLG values. The results were compared with the measured data. Optimal DLG values were determined using global gamma criteria (3%/2 mm and 2%/2 mm), local gamma (1%/1 mm), and dose difference criteria.

**Results:**

Verification with radiochromic film using IC‐based optimal DLG values yielded an average GPR of 98.7 ± 2.3% under the 3%/3 mm criterion. The average DLG difference between the IC and diode array dosimeters was −0.35 ± 0.23 mm (range: −0.80 to 0.26 mm), based on various gamma and dose difference criteria. A DLG difference of −0.35 mm showed dose deviations ranging from 0.27% to 1.05% across all beams. Except for one criterion, no significant differences were observed.

**Conclusions:**

DLG differences between IC and diode array dosimeters resulted in approximately 1% dose discrepancies, which are within the clinically acceptable range. This finding indicates that ArcCHECK, Delta4, and SRS MapCHECK can determine DLG values comparable to those obtained with IC.

## 1 INTRODUCTION

The dosimetric leaf gap (DLG) value is a key parameter in the Varian Eclipse treatment planning system (TPS). This parameter represents a geometric offset that accounts for transmission using rounded multileaf collimator (MLC) leaf tips during dose calculation. Inaccurate DLG settings can result in clinically significant dose discrepancies. However, this parameter is barely revisited after the initial commissioning.^1^, ^2^, ^3^, ^4^, ^5^ Although similar modeling concepts exist in other TPSs, the explicit use of a single DLG parameter is unique to Eclipse.

DLG contributes to advanced treatment techniques, such as volumetric modulated arc therapy (VMAT), intensity‐modulated radiation therapy (IMRT), and stereotactic radiotherapy, where precise modeling of narrow leaf gaps and complex MLC motion is essential. Traditionally, DLG is measured using the sweeping gap method with an ionization chamber (IC) and film,^3^, ^6^, ^7^ or using other detectors such as electronic portal imaging devices (EPIDs) and optically stimulated luminescent dosimeters.^8^, ^9^ Among these, ICs are widely used for DLG measurements owing to their high accuracy, reproducibility, and minimal directional dependence. Their reliability has been demonstrated in several commissioning and validation studies, including AAPM Task Group 119 (TG‐119).^10^ Accordingly, in this study, IC‐based DLG values were used as the reference standard for comparison with diode array dosimeters.

Measured DLG values may not always yield optimal agreement with the delivered doses in complex clinical plans due to limitations in the TPS modeling. Therefore, plan‐based optimization of DLG value—adjusting DLG value within the TPS and verification against measured doses from actual clinical plans—is an essential process for ensuring accurate dose calculation.^11^, ^12^, ^13^ Previous studies, including the multi‐center analysis by Isono et al.,^14^ have shown that institutions frequently adjust DLG value (typically upward) and leaf transmission factor (LTF) values based on clinical plan QA results.

Among the available QA devices, diode array dosimeters, such as ArcCHECK (Sun Nuclear), Delta4 (ScandiDos), and SRS MapCHECK (Sun Nuclear) are widely used to adjust the optimal DLG value. These devices are convenient and fast, providing instant readout of planar dose distributions rather than single‐point measurements. These devices can measure composite planar dose distributions while accounting for gantry rotation and couch absorption. Moreover, they support gamma index analysis to quantify the agreement with TPS calculations. A typically used metric is the gamma pass rate (GPR), with the AAPM TG‐218 recommending tolerance criteria of 95% (action level 90%) using a 3% dose difference and 2 mm distance to agreement (3%/2 mm). However, GPR can vary among devices, even when analyzing the same treatment plan, due to differences in the measurement planes and algorithm implementations. For example, up to a 6% variation in GPR has been reported across devices in TG‐218.^15^

Therefore, this study evaluates whether the optimal DLG values obtained using ArcCHECK, Delta4, and SRS MapCHECK are comparable to those obtained using IC measurements under clinically relevant plan verification conditions.

## 2 MATERIALS AND METHODS

Five representative cases were selected for each beam energy, resulting in a total of twenty plans. The selected cases reflect typical clinical scenarios typically treated at our institution for each energy: 6 and 10 MV photon beams, and 6 and 10 MV flattening filter‐free (FFF) photon beams. In particular, 6 and 10 MV beams are used in VMAT plans except for stereotactic body radiotherapy (SBRT), while 6 and 10 MV FFF are primarily used for SBRT. Detailed information regarding the treatment site, target volume size, number of arcs, field size, and monitor units are presented in Table 1. The treatment unit was TrueBeamSTx (Varian Medical Systems, Palo Alto, CA, USA) equipped with a high‐definition multi‐leaf collimator (HD‐MLC). A non‐clinical version of the TPS, Eclipse version 16.1 (Varian Medical Systems, Palo Alto, CA, USA) was used for this study. The TPS settings included spot sizes in the X and Y directions of 1 mm and 0.5 mm, respectively. The LTFs for 6 MV, 10 MV, 6 MV FFF, and 10 MV FFF were set at 1.1%, 1.4%, 1.1%, and 1.2%, respectively. These values were measured and optimized for LTF at our institution, thereby reflecting the specific parameters of our institution. Acuros XB was used as the calculation algorithm. At our institution, the IMRT dose output of selected photon beam energy was independently verified by a third‐party organization within our country, and confirmed to be within clinically acceptable limits.

**TABLE 1:** Detailed information on treatment plans used for verification.

					Field 1		Field 2		
Energy (MV)	Site and definition name	PTV volume (cc)	No. of arcs	Fractionation	X jaw size (cm)	Y jaw size (cm)	X jaw size (cm)	Y jaw size (cm)	MU
6	Lung 1	47.7	2	2.00 Gy × 30 Fr	5.8	6.4	5.8	6.4	259, 444
	Lung 2	591.4	2	1.50 Gy × 20 Fr	11.0	16.0	11.0	16.0	349, 389
	H&N 1	459.4	2	2.00 Gy × 35 Fr	13.7	18.9	13.7	18.9	296, 324
	H&N 2	681.7	2	2.00 Gy × 35 Fr	14.7	17.5	14.9	17.5	315, 283
	Head	765.8	1	2.00 Gy × 23 Fr	13.6	14.0	–	–	515
10	Prostate 1	88.3	1	2.00 Gy × 37 Fr	7.1	7.5	–	–	567
	Prostate 2	77.0	1	3.10 Gy × 20 Fr	6.6	6.6	–	–	1077
	Pelvis 1	1743.2	2	2.40 Gy × 25 Fr	15.0	22.0	15.0	22.0	347, 293
	Pelvis 2	1836.5	2	1.80 Gy × 20 Fr	15.0	22.0	15.0	22.0	290, 276
	Esophagus	200.7	2	2.00 Gy × 30 Fr	9.5	17.7	9.5	17.7	246, 235
6 FFF	Lung 1	15.6	2	7.50 Gy × 8 Fr	4.5	4.5	4.6	4.4	1142, 1171
	Lung 2	7.3	2	7.50 Gy × 8 Fr	2.9	3.1	3.2	2.9	1164, 976
	Lung 3	9.7	2	12.00 Gy × 5 Fr	4.0	3.7	4.0	3.7	1869, 1921
	Lung 4	9.0	1	10.00 Gy × 5 Fr	3.8	4.1	–	–	3324
	Lung 5	53.1	2	10.00 Gy × 5 Fr	5.4	2.7	6.0	5.3	1806, 1888
10 FFF	Prostate 1	67.7	2	7.25 Gy × 5 Fr	7.5	6.7	7.5	6.7	1600, 1378
	Prostate 2	36.7	1	7.25 Gy × 5 Fr	5.6	5.8	–	–	2348
	Spine 1	17.1	2	9.00 Gy × 3 Fr	4.6	4.4	3.6	4.3	1504, 1371
	Spine 2	144.1	2	9.00Gy × 3 Fr	7.6	10.0	10.0	10.0	1800, 2321
	Ilium	107.7	1	5.00 Gy × 7Fr	10.5	10.4	–	–	1583

*Note*: “Field 1” and “Field 2” refer to the two arcs in a dual‐arc volumetric modulated arc therapy (VMAT) plan. “Site and definition name” indicates the anatomical treatment site and the specific plan identifier in the treatment planning system. “No. of arcs” represents the total number of arcs in the VMAT plan. “Fractionation” is presented dose per fraction in Gy and the total number of fractions. “X jaw size” and “Y jaw size” denote the collimator jaw positions in the X and Y directions at the isocenter plane.

Abbreviations: FFF, Flattening filter‐free; H&N, Head and neck; MU, monitor unit; cc, cubic centimeters; PTV, Planning target volume.

We used three diode array dosimeters: ArcCHECK, Delta4, and SRS MapCHECK. A CC13 cylindrical IC (IBA Dosimetry, Schwarzenbruck, Germany) was also used. Moreover, Gafchromic EBT3 and XD films (Ashland Advanced Materials, Bridgewater, NJ, USA) were employed to verify the optimal DLG values determined using IC. EBT3 films were utilized at 6 and 10 MV, whereas XD films were utilized for 6 and 10 MV FFF verifications. Gafchromic films were scanned using an Epson ES‐G11000 flatbed scanner (Seiko Epson Corp., Japan) in the transmission mode at 150 dpi. Further image processing and dose calibration were performed using the DD‐System(R‐TECH, Japan). ArcCHECK comprised 1386 diodes arranged in a curved array in a 26.6 cm diameter cylindrical polymethyl methacrylate (PMMA) phantom. The detectors were arranged spirally 1 cm apart along a cylindrical length and circumference spacing of 1 cm.^16^ Delta4 contained 1069 diodes on two orthogonal boards. The detectors were spaced at 0.5 cm in each board within the central 6 × 6 cm^2^ region and 1 cm elsewhere in the 20 × 20 cm^2^ active area. The boards were inserted into a 22 cm diameter PMMA phantom.^16^, ^17^ SRS MapCHECK comprised 1013 diodes on one board. The detector was spaced 2.47 mm within a 77 × 77 mm^2^ active area.^18^, ^19^ Initially, VMAT plans using 6 and 10 MV were measured with ArcCHECK and Delta4, while those using 6 and 10 MV FFF were measured with ArcCHECK, Delta4, and SRS MapCHECK. Moreover, all VMAT plans were measured using an IC inserted in the center of I'mRT phantom (IBA Dosimetry; Schwarzenbruck, Germany). Radiochromic films were inserted in a horizontal orientation at the mid‐plane of the I'mRT phantom.

Each phantom was set up at the iso‐center for the ArcCHECK, Delta4, SRS MapCHECK, IC, and film measurements. The IC was moved to an appropriate position so that the dose in the target could be measured when the dose was set in a low‐dose area. First, dose calibration was performed to eliminate the effect of daily output fluctuations of the linear accelerator on the diode array and IC measurements. Subsequently, all initial plans were measured using all verification tools. In the TPS, the plans were recalculated in the TPS by adjusting the DLG from 0.4 to 2.2 mm in increments of 0.2 mm. Considering the time and resource constraints associated with each measurement, this sample size was deemed appropriate and feasible for the study purposes. The calculation grid sizes were 1.5 mm for analyses involving ArcCHECK, Delta4, and IC, and 1 mm for SRS MapCHECK, as recommended for these tools. These doses were compared with the those measured using the ArcCHECK, Delta4, SRS MapCHECK, and IC. For diode array dosimeters, the dose distributions were compared using diode‐array‐dosimeter software to analyze the GPR and dose differences. The DLG yielding the highest GPR was designated as the optimal DLG for each case (the average of the DLG values obtained when two or three points shared the highest GPR). The average of these values across cases was defined as the optimal DLG. The analysis evaluated the GPR using both global and local normalization. In global normalization, the dose difference between all measured and calculated dose point pairs was normalized using the maximum planned dose point. In the local normalization, the dose difference for all point pairs was normalized to the planned dose at the local point. Global GPR was assessed for 3%/2 mm and 2%/2 mm. In addition, the local GPR was evaluated to be 1%/1 mm. The gamma analysis considered only dose points receiving more than 10% of the maximum dose. The gamma criterion of 3%/2 mm global was selected and is referred to as TG‐218.^15^ Moreover, 2%/2 mm global and 1%/1 mm local were selected to evaluate the distribution similarities in more detail. In addition to gamma analysis, a simple dose difference was evaluated. Two different metrics were used to evaluate the dose differences. For ArcCHECK and SRS MapCHECK, the dose difference was defined as the average difference between the measured and planned doses at all measurement points. For Delta4, the dose difference was defined as the median percentage difference between the measured and planned doses at each dose point within the region receiving more than 20% of the maximum dose. The optimal DLG was defined as either the value that resulted in the highest GPR or the DLG corresponding to a zero‐dose error derived from the linear approximation between the dose difference and DLG. For the IC, the optimal DLG was determined as the value at which the dose difference between the IC and planned dose reached zero, based on the linear approximation line between the dose difference and the DLG. The optimal DLG was determined for each plan and averaged to obtain the optimal DLG for each energy. For the verification using radiochromic films, after normalizing the absolute dose at an appropriate location, gamma analysis was conducted using the criteria of 3%/3 mm global GPR with a threshold of 10% of the maximum dose in accordance with the AAPM‐119.^10^

The differences in optimal DLG values determined using ArcCHECK and Delta4 were evaluated for 6 and 10 MV photon beams based on gamma criteria and dose difference, with IC measurements used as the reference standard. In contrast, differences in optimal DLG values obtained from all tools (ArcCHECK, Delta4, and SRS MapCHECK) were assessed for 6 and 10 MV FFF beams, also referring to IC measurements.

A two‐sided Wilcoxon signed‐rank test was used to determine the differences in the measured optimal DLG values of the diode array dosimeters and ICs. We applied the Holm‐Bonferroni method to control the family‐wise error rate across the eight Wilcoxon tests for each device. In addition to the *p*‐values, effect sizes (*r*) were calculated to quantify the magnitude of the differences. The effect size “*r*” was calculated from the *z*‐score as *r* = Z/√N, where N is the number of pairs. Smaller effect sizes indicate higher agreement between the systems. The agreement level was based on effect size: high (|*r*| <0.3), moderate (0.3 ≤ |*r*| < 0.5), and low (|*r*| ≥ 0.5). Statistical significance was set at *p* < 0.05. Statistical analyses were performed using IBM SPSS Statistics (version 26.0; IBM Corp., Armonk, NY, USA).

## 3 RESULTS

**FIGURE 3:**
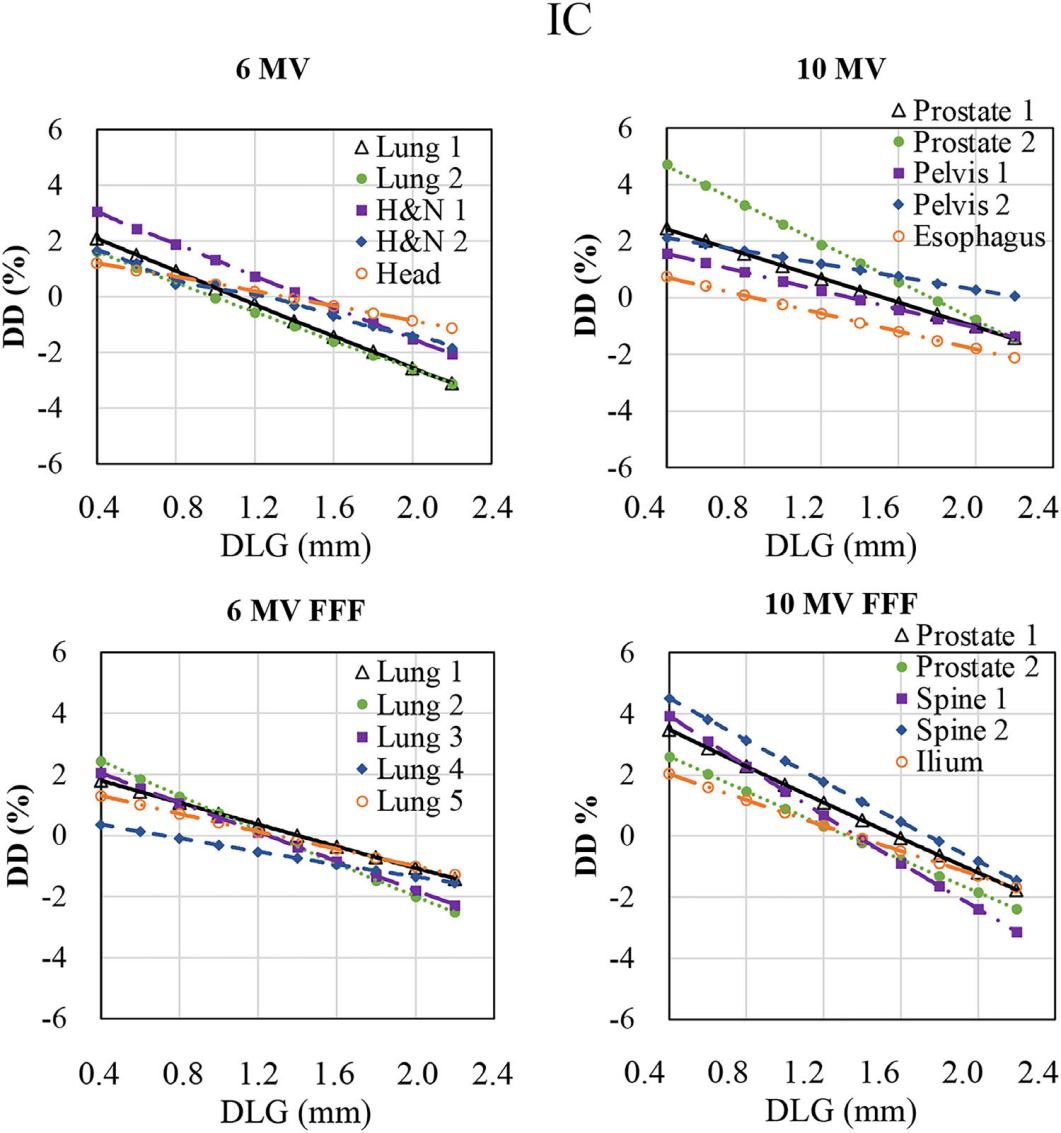
Relationship between an ionization chamber (IC) dose difference (DD) and dosimetric leaf gap (DLG) changes. The *x*‐axis represents the change in DLG values, whereas the y‐axis shows the corresponding change in DD between IC measurements and TPS calculations. Measurements were conducted using an IC with clinical volumetric‐modulated arc therapy plans.

**FIGURE 4:**
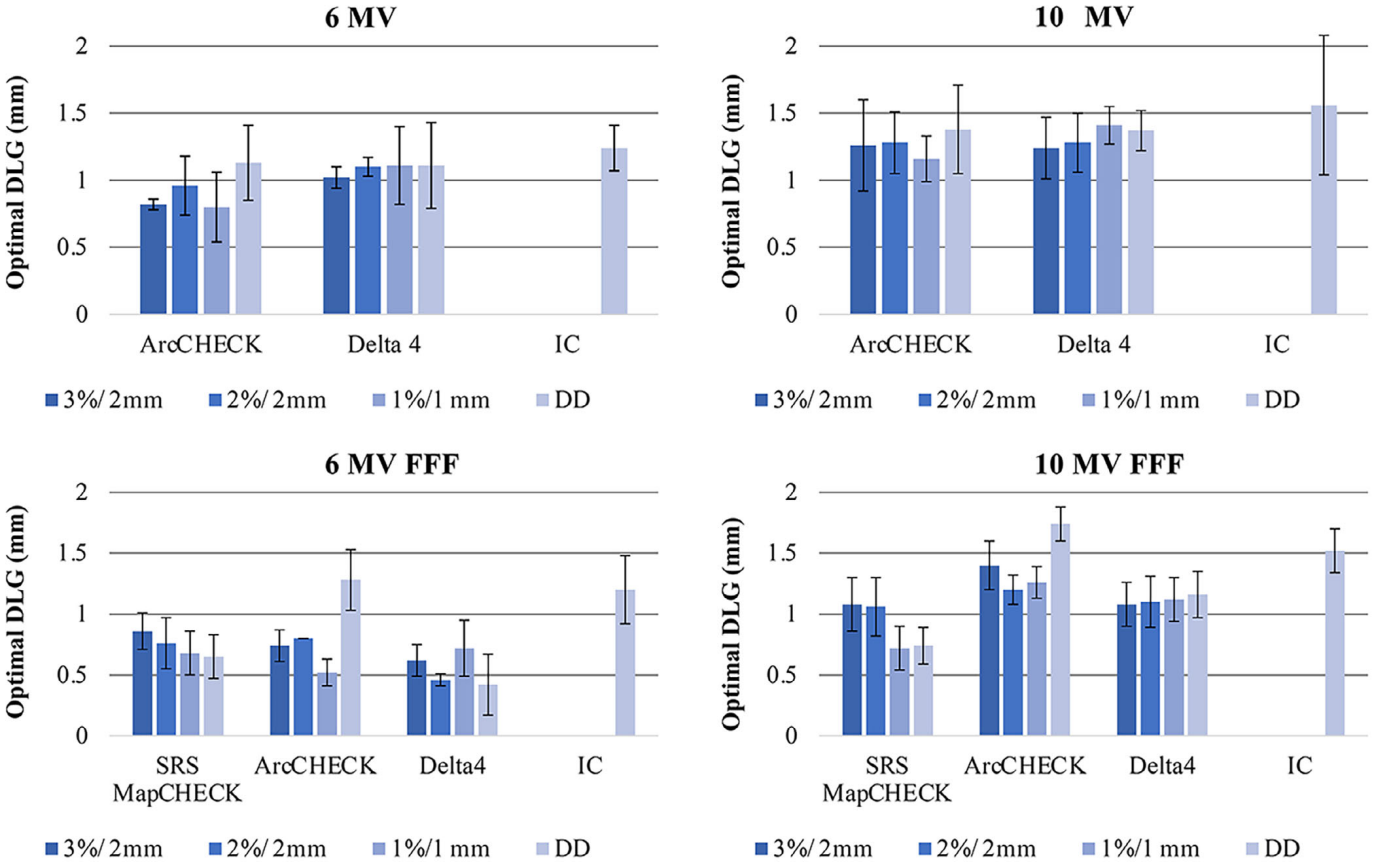
Comparison of optimal dosimetric leaf gap (DLG) values across all energies and tools. The 3%/2 mm and 2%/2 mm showed optimal DLG values according to the global gamma criterion, whereas the 1%/1 mm showed optimal DLG values according to the local gamma criterion. The DD indicates the optimal DLG values according to the dose difference between the measured and calculated doses.

Table 2 summarizes the differences in optimal DLG values between each diode array detector and the IC. In contrast, Figures 1, 2, 3 illustrate the variations in GPR and dose difference as a function of the DLG values for each diode array detector and beam energy. Figure 4 shows a comparison of the optimal DLG values derived across all tools and energies. Table 3 summarizes the comparison of the dose distributions obtained based on the optimal DLG values determined by IC measurements with those obtained from radiochromic film measurements. All treatment plans, except for the esophageal plan, achieved GPRs exceeding 95% with the 3%/3 mm criterion. The values measured with the dose difference for ArcCHECK optimal DLG values of 6 and 10 MV FFF were slightly higher than those obtained by IC. In contrast, the other values were slightly lower than those obtained using IC. The mean and standard deviation of the difference between the optimal DLG measured with the diode array dosimeters and the DLG determined by IC was −0.35 ± 0.23 mm (range: −0.80 to 0.26 mm). A linear approximation can be assumed for each plan based on the relationship between the DLG and the dose error from the IC measurements. Therefore, the effect of DLG errors on the dose was calculated from the straight lines shown in Figure 3 for 6 MV, 10 MV, 6 MV FFF, and 10 MV FFF. Consequently, a DLG difference of −0.35 mm resulted in dose deviations of 0.80%, 0.70%, 0.27%, and 1.05% for 6 MV, 10 MV, 6 MV FFF, and 10 MV FFF IC measurements, respectively. In the statistical comparison test, before applying the Holm‐Bonferroni correction, slight significant differences in optimal DLG were observed for 6 and 10 MV. In contrast, significant differences were observed for 6 and 10 MV FFF across several evaluation criteria. However, after applying the Holm‐Bonferroni correction, most of the differences were not statistically significant. Only one comparison—SRS MapCHECK at 10 MV FFF under the 1%/1 mm criterion—remained significant. Therefore, the results indicate that no statistically significant differences in the optimal DLG existed among the devices after correction. However, for the 6 and 10 MV, the agreement was more sensitive to the stricter criteria of 2%/2 mm, 1%/1 mm, and dose difference than to the 3%/3 mm criteria. In contrast, for the 6 and 10 MV FFF, no notable agreement was observed across the various gamma pass criteria.

**TABLE 2:** Difference in optimal dosimetric leaf gap (DLG) values measured by diode array dosimeters and ionization chamber (IC).

Dosimeter	Energy (MV)	Criteria	DLG (Diode) (mm)	DLG (IC) (mm)	Difference (mm)	*p*‐value	Z	Adjusted *p*	Effect size r	Agreement
ArcCHECK	6	3%/ 2mm	0.82 ± 0.04	1.24±0.17	−0.42	0.04*	−2.02	0.34	0.72	Low
		2%/ 2mm	0.96 ± 0.22		−0.28	0.23	−1.21	0.90	0.43	Moderate
		1%/1 mm	0.80 ± 0.26		−0.44	0.04*	−2.02	0.30	0.72	Low
		DD (Average)	1.13 ± 0.28		−0.11	0.50	−0.67	1.00	0.24	High
	10	3%/2mm	1.26 ± 0.34	1.56±0.52	−0.30	0.08	−1.75	0.48	0.62	Low
		2%/ 2mm	1.28 ± 0.23		−0.28	0.50	−0.67	1.00	0.24	High
		1%/1 mm	1.16 ± 0.17		−0.40	0.08	−1.75	0.40	0.62	Low
		DD (Average)	1.38 ± 0.33		−0.18	0.89	−0.14	0.89	0.05	High
Delta 4	6	3%/ 2mm	1.02±0.08	1.24±0.17	−0.22	0.14	−1.48	1.00	0.52	Low
		2%/ 2mm	1.10 ± 0.07		−0.14	0.23	−1.21	1.00	0.43	Moderate
		1%/1 mm	1.11±0.29		−0.13	0.35	−0.94	1.00	0.33	Moderate
		DD (Median)	1.11±0.32		−0.13	0.50	−0.67	1.00	0.24	High
	10	3%/ 2mm	1.24±0.23	1.56±0.52	−0.32	0.14	−1.48	0.97	0.52	Low
		2%/ 2mm	1.28±0.22		−0.28	0.14	−1.48	0.83	0.52	Low
		1%/1 mm	1.41±0.14		−0.15	0.50	−0.67	0.50	0.24	High
		DD (Median)	1.37±0.15		−0.19	0.35	−0.94	1.00	0.33	Moderate
SRS MapCHECK	6 (FFF)	3%/ 2mm	0.86±0.15	1.20±0.28	−0.34	0.04*	−2.03	0.25	0.72	Low
		2%/ 2mm	0.76±0.21		−0.44	0.04*	−2.04	0.33	0.72	Low
		1%/1 mm	0.68±0.18		−0.52	0.04*	−2.04	0.29	0.72	Low
		DD (Average)	0.65±0.18		−0.55	0.04*	−2.02	0.17	0.72	Low
	10 (FFF)	3%/ 2mm	1.08±0.22	1.52±0.18	−0.44	0.04*	−2.02	0.13	0.72	Low
		2%/ 2mm	1.06±0.24		−0.46	0.04*	−2.02	0.09	0.72	Low
		1%/1 mm	0.72±0.18		−0.80	0.04*	−2.02	0.04*	0.72	Low
		DD (Average)	0.74±0.15		−0.78	0.04*	−2.03	0.21	0.72	Low
ArcCHECK	6 (FFF)	3%/ 2mm	0.74±0.13	1.20±0.28	−0.46	0.04*	−2.02	0.17	0.72	Low
		2%/ 2mm	0.80±0.00		−0.40	0.04*	−2.07	0.30	0.73	Low
		1%/1 mm	0.52±0.11		−0.68	0.04*	−2.04	0.29	0.72	Low
		DD (Average)	1.28±0.25		0.08	0.89	−0.14	0.89	0.05	High
	10 (FFF)	3%/ 2mm	1.40±0.20	1.52±0.18	−0.12	0.04*	−2.03	0.25	0.72	Low
		2%/ 2mm	1.20±0.12		−0.32	0.04*	−2.03	0.21	0.72	Low
		1%/1 mm	1.26±0.13		−0.26	0.07	−1.84	0.20	0.65	Low
		DD (Average)	1.74±0.14		0.22	0.07	−1.83	0.14	0.65	Low
Delta4	6 (FFF)	3%/ 2mm	0.62±0.13	1.20±0.28	−0.58	0.04*	−2.03	0.25	0.72	Low
		2%/ 2mm	0.46±0.05		−0.74	0.04*	−2.04	0.33	0.72	Low
		1%/1 mm	0.72±0.23		−0.48	0.04*	−2.04	0.29	0.72	Low
		DD (Median)	0.42±0.25		−0.78	0.04*	−2.02	0.22	0.72	Low
	10 (FFF)	3%/ 2mm	1.08±0.18	1.52±0.18	−0.44	0.04*	−2.02	0.17	0.72	Low
		2%/ 2mm	1.10±0.21		−0.42	0.04*	−2.02	0.13	0.72	Low
		1%/1 mm	1.12±0.18		−0.40	0.06	−1.86	0.13	0.66	Low
		DD (Median)	1.16±0.19		−0.36	0.07	−1.84	0.07	0.65	Low

*Note*: Adjusted *p*‐value adjusted for multiple comparisons using the Holm‐Bonferroni method.

The Global gamma passing rate (GPR) was evaluated using the 3%/2mm and 2%/2mm criteria, whereas the Local GPR was evaluated using the 1%/1mm criterion. DLG values are presented as mean ± standard deviation. The “Difference” column represents the absolute difference between DLG (Diode) and DLG (IC). Statistical comparisons between the two methods were performed using the Wilcoxon signed‐rank test, which provided the *z*‐score and *p*‐value. The effect size “*r*” was calculated from the *z*‐score as *r* = Z/√N, where N is the number of pairs. The agreement level was based on effect size: High (|*r*| <0.3), Moderate (0.3 ≤ |*r*| < 0.5), and Low (|*r*| ≥0.5).

Abbreviations: FFF, flattening filter‐free; DLG (Diode), Optimal DLG values using diode array dosimeters; DLG (IC), Optimal DLG values using IC; DD (Average), mean percentage dose difference; DD (Median), median percentage dose difference.

*p < 0.05

FIGURE 1Relationship between the change in gamma pass rate (GPR) and the dose difference (DD) and the change in dosimetric leaf gap (DLG) for ArcCHECK. (6 MV, 3%/2 mm and 2%/2 mm global GPR, 1%/1 mm local GPR, and dose difference). The *x*‐axis represents the change in DLG values, whereas the *y*‐axis shows the corresponding change in GPR or DD. Measurements were conducted using ArcCHECK with clinical volumetric‐modulated arc therapy (VMAT) plans. (a) Relationship between the change in GPR and the DD, and the change in DLG for Delta4. (6 MV, 3%/2 mm and 2%/2 mm global GPR, 1%/1 mm local GPR, and dose difference). The *x*‐axis represents the change in DLG values, whereas the *y*‐axis shows the corresponding change in the GPR or DD. Measurements were conducted using Delta4 with clinical VMAT plans. (b) Relationship between the change in GPR and the DD, and the change in DLG for ArcCHECK. (10 MV, 3%/2 mm and 2%/2 mm global GPR, 1%/1 mm local GPR, and DD). The *x*‐axis represents the change in DLG values, whereas the *y*‐axis shows the corresponding change in the GPR or DD. Measurements were conducted using ArcCHECK with clinical VMAT plans. (c) Relationship between the change in GPR and DD and the change in DLG for ArcCHECK. (10 MV, 3%/2 mm and 2%/2 mm global GPR, 1%/1 mm local GPR, and DD). The *x*‐axis represents the change in DLG values, whereas the *y*‐axis shows the corresponding change in the GPR or DD. Measurements were conducted using Delta4 with clinical VMAT plans.

**Figure:**
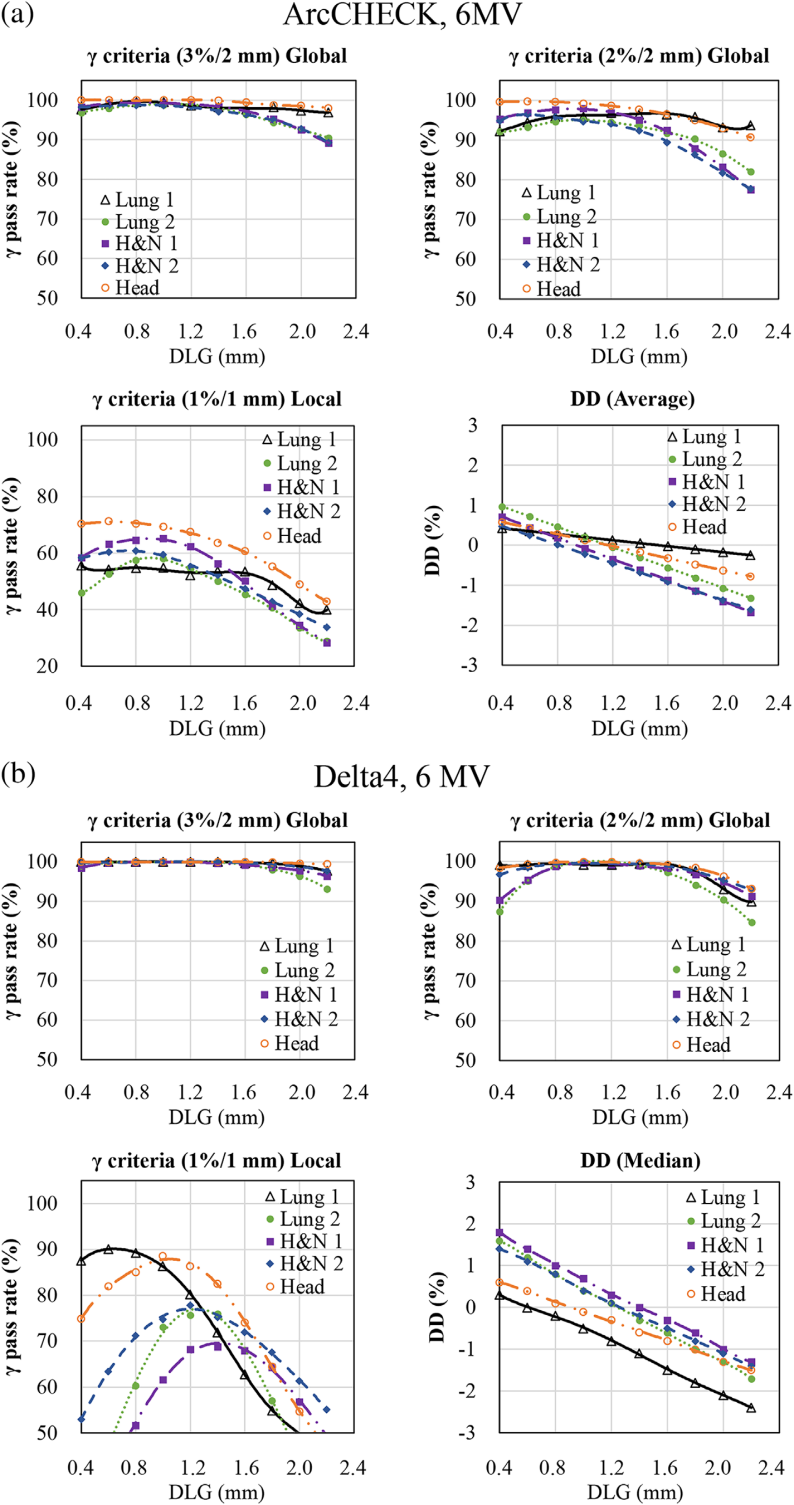

**Figure:**
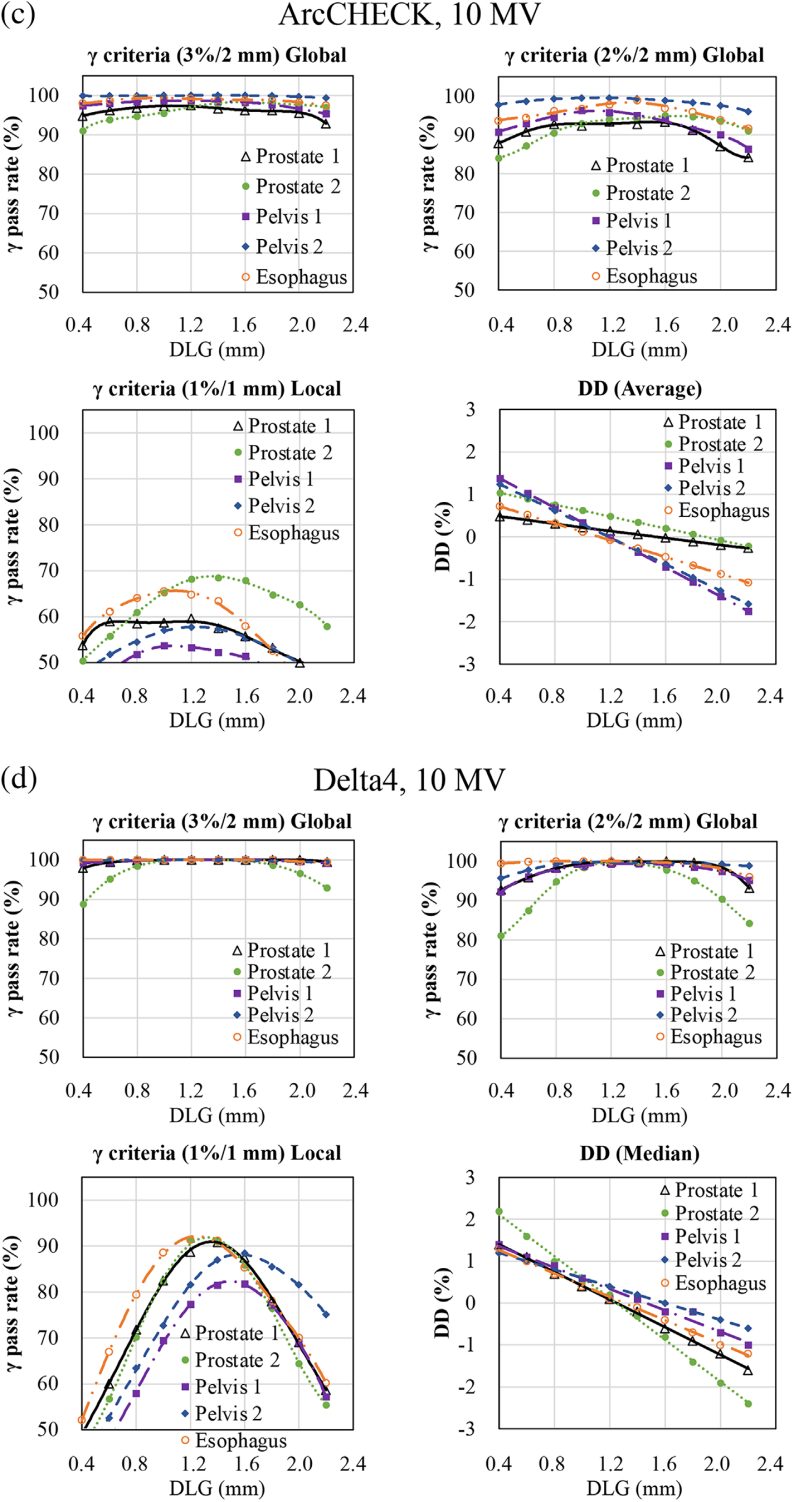

FIGURE 2(a) Relationship between the change in gamma pass rate (GPR) and the dose difference (DD) and the change in dosimetric leaf gap (DLG) for SRS MapCHECK, ArcCHECK, and Delta4. (6 MV FFF, 3%/2 mm and 2%/2 mm global GPR). The x‐axis represents the change in DLG values, whereas the y‐axis shows the corresponding change in the GPR or DD. Measurements were conducted using three diode array dosimeters with clinical volumetric‐modulated arc therapy (VMAT) plans. (b) Relationship between the change in GPR and DD and the change in DLG for SRS MapCHECK, ArcCHECK, and Delta4. (6 MV FFF, 1%/1 mm local GPR and DD). The x‐axis represents the change in DLG values, whereas the y‐axis shows the corresponding change in the GPR or DD. Measurements were conducted using three diode array dosimeters with clinical VMAT plans. (c) Relationship between the change in GPR and DD and the change in DLG for SRS MapCHECK, ArcCHECK, and Delta4. (10 MV FFF, 3%/2 mm and 2%/2 mm global GPR). The x‐axis represents the change in DLG values, whereas the y‐axis shows the corresponding change in the GPR or DD. Measurements were conducted using three diode array dosimeters with clinical VMAT plans. (d) Relationship between the change in GPR and DD and the change in DLG for SRS MapCHECK, ArcCHECK, and Delta4. (10 MV FFF, 1%/1 mm local GPR and DD). The *x*‐axis represents the change in DLG values, whereas the y‐axis shows the corresponding change in the GPR or DD. Measurements were conducted using three diode array dosimeters with clinical VAMT plans.

**Figure:**
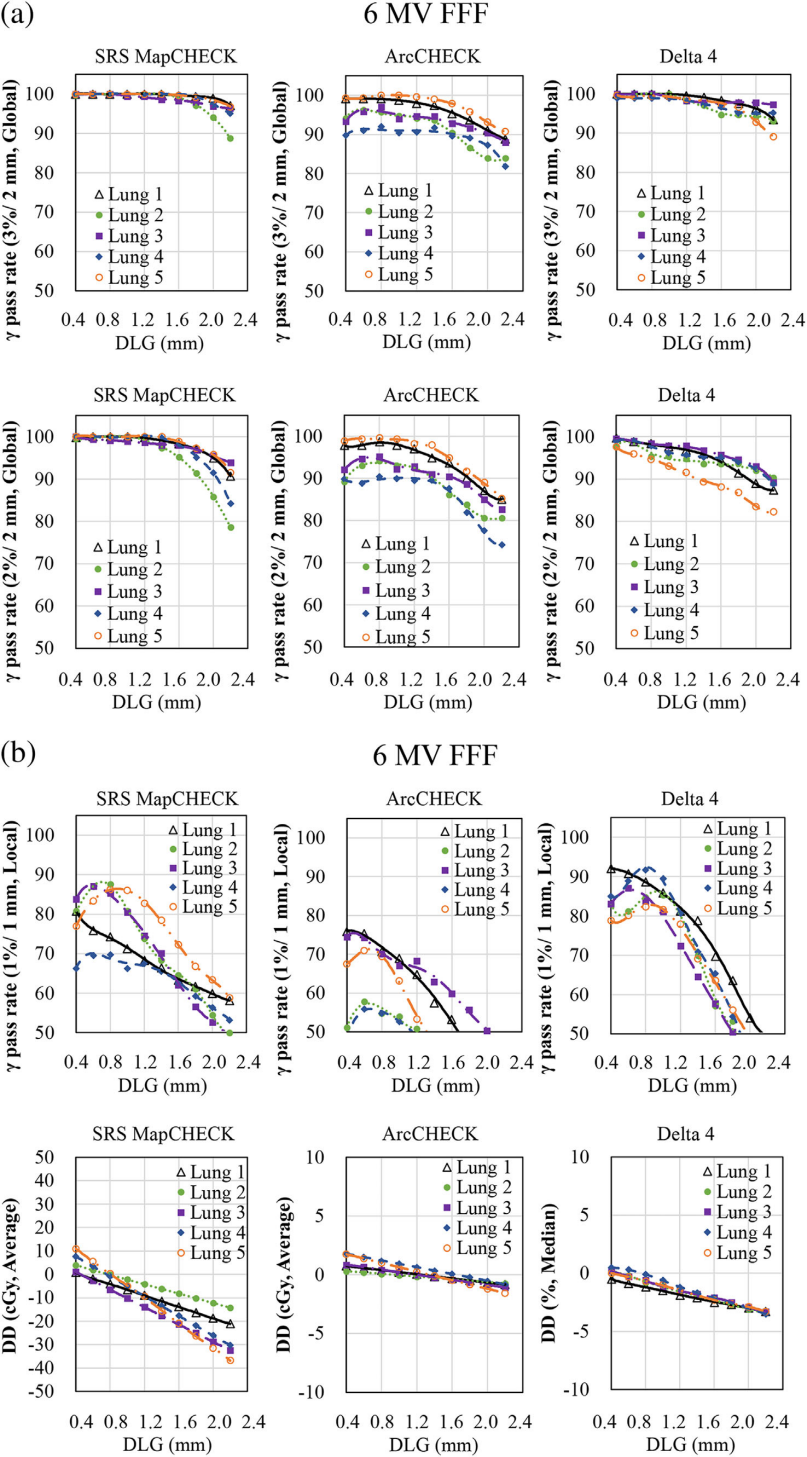

**Figure:**
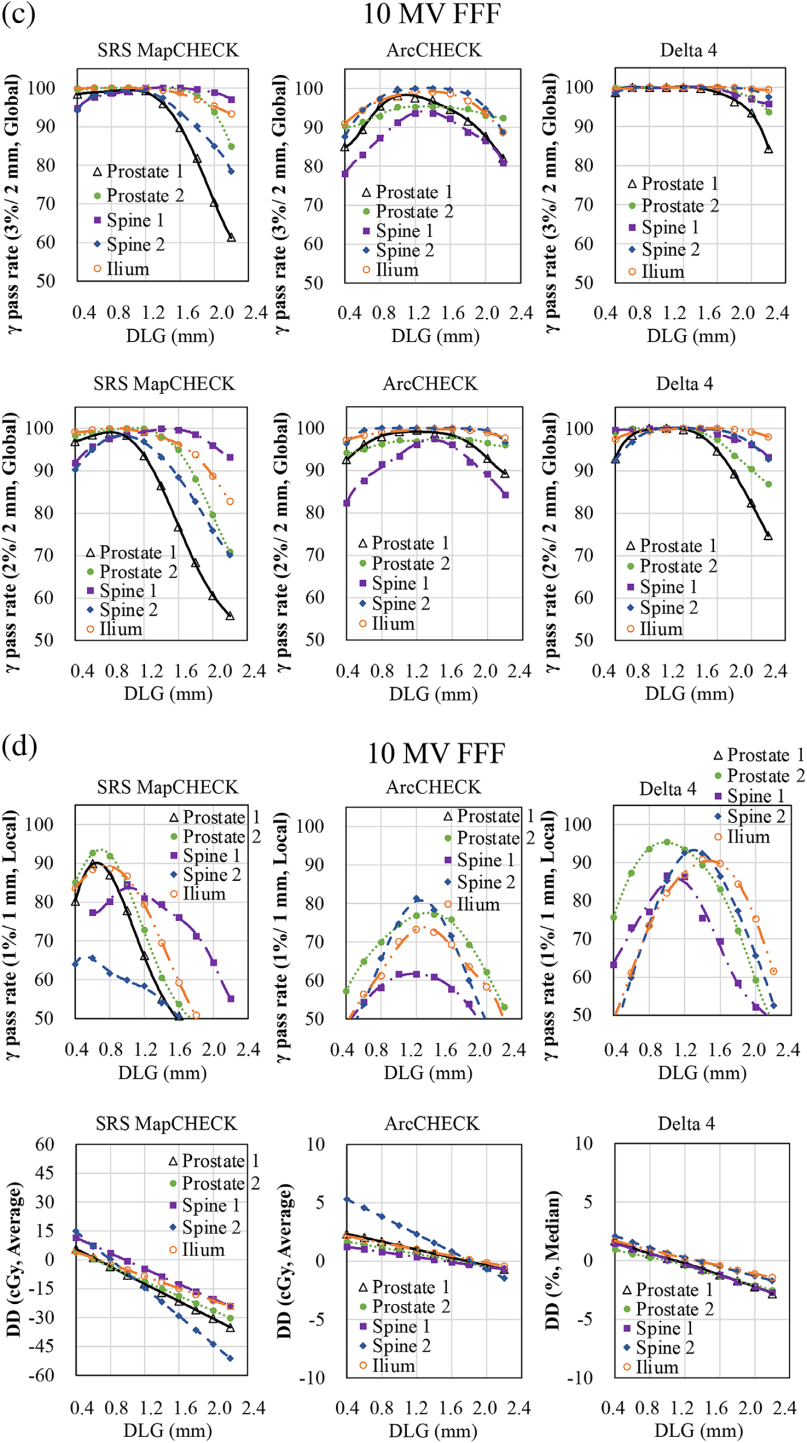

**TABLE 3:** Summary of gamma passing rates (3%/3 mm) for radiochromic films, evaluated using optimal dosimetric leaf gap values derived from ionization chamber measurements.

Energy (MV)	6					10				
Site and definition name	Lung 1	Lung 2	Head	H&N 1	H&N 2	Prostate 1	Prostate 2	Pelvis 1	Pelvis 2	Esophagus
Gamma passing rate (%)	99.9	100	95.0	97.7	99.8	100	100	95.0	95.4	92.8

Energy (MV)	6 FFF					10 FFF				
Site and definition name	Lung 1	Lung 2	Lung 3	Lung 4	Lung 5	Prostate 1	Prostate 2	Spine 1	Spine 2	Ilium
Gamma passing rate (%)	100	100	100	100	100	100	100	100	100	99.2

Abbreviations: FFF, Flattening filter‐free; H&N, Head and neck.

## 4 DISCUSSIONS

The DLG values used in Eclipse TPS are determined using ICs, films, EPIDs, and other devices.^3^, ^6^, ^7^, ^8^, ^9^, ^14^, ^15^ Moreover, some institutions employ a diode array dosimeter because they are convenient and are used regularly in patient‐specific QA.^14^ However, the GPR varies depending on the diode array dosimeters, even when the same plan is verified.^15^ In particular, whether different diode array dosimeters provide consistent DLG measurements in radiotherapy TPS has not yet been clarified. Thus, this study aimed to obtain optimal DLG values using ArcCHECK, Delta4, and SRS MapCHECK, and determine whether significant differences arise when the obtained values are compared with those obtained by IC, which is considered the reference standard.

Variability in optimal DLG values was observed across different treatment sites when measured using IC. The standard deviations of the optimal DLG for the 6 MV, 10 MV, 6 MV FFF, and 10 MV FFF were 0.19, 0.48, 0.28, and 0.16 mm, respectively. This variability is likely attributable to differences in MLC leaf trajectories and aperture patterns among individual treatment sites. The film analysis using the optimal DLG values measured with the IC demonstrated GPRs exceeding 95% (3%/3 mm) for all cases except for one. These results support the validity of the IC‐based DLG values, as GPRs above 95% under the 3%/3 mm criteria are generally considered acceptable and align with the benchmark outcomes reported in AAPM TG‐119.^10^ Notably, the film analysis of the esophageal plan with 10 MV resulted in a GPR of 92.8% with the same criteria, which was slightly lower than expected. This may be attributed to the larger standard deviation observed for 10 MV and reflects the fact that the optimal DLG value for the esophageal plan slightly differed from those of other treatment sites. Because film dosimetry involves normalization to the appropriate point dose, it cannot be used to determine the optimal DLG values directly. In this study, the film was used to verify that the DLG values determined by IC measurements produced acceptable dose distributions rather than as a tool for optimizing DLG.

The measurement results showed that the average difference between the optimal DLG measured with the diode array dosimeters and IC were −0.35 ± 0.23 mm (range: −0.80 to 0.26 mm), which corresponds to a dose of 0.80%, 0.70%, 0.27%, and 1.05% by IC measurements (6 MV, 10 MV, 6 MV FFF, and 10 MV FFF, respectively). Given the variability among individual cases and considering that ICs measure the dose at a single point—introducing minor positional uncertainties—these differences are relatively small. A dose accuracy of ±5% is generally acceptable in VMAT (as stated in ICRU Report 83^20^ and AAPM TG‐119^10^); therefore, a discrepancy of approximately 1% from DLG variation is considered clinically tolerable. The observed discrepancies can be attributed to the differences in the measurement characteristics. First, the IC measures the dose at a single point, which may not fully capture spatial dose variations. Second, additional corrections are applied to the diode array dosimeters, including adjustments for energy and angular dependence. Although these correction factors are generally effective in compensating for the inherent limitations of diodes, they may not fully replicate IC‐based measurements under all conditions. These factors likely contribute to the observed discrepancies in IC‐based DLG values. However, the magnitude of these differences is small and remains within clinically acceptable limits, demonstrating that the clinical impact is negligible.

In this study, a global GPR of 3%/2 mm was used as a reference for TG‐218,^15^ and a tighter global GPR of 2%/2 mm, local passing rate of 1%/1 mm, and the dose differences were also used as indices to determine the optimal DLG. A trend observed with both 6 and 10 MV was that better agreement was achieved when using stricter criteria, such as 2%/2 mm, 1%/1 mm, and dose difference, compared to 3%/2 mm gamma criteria with 6 and 10MV. This suggests that applying tighter constraints or using dose difference metrics results in a higher level of agreement between the optimal DLG values measured using the IC and those derived from diode array dosimeters. This trend was not observed with 6 and 10 MV FFF. As shown in Figures 1 and 2, the relationship between DLG and GPR becomes relatively flat under looser criteria. Consequently, the optimal DLG value is difficult to determine. These findings emphasize that selecting a DLG solely because it achieves a GPR exceeding 95% under a lenient threshold, such as 3%/2 mm, is inappropriate as this result may conceal errors that would be detected using stricter evaluation criteria.

Yao and Farr^21^ proposed a method to determine optimal DLG values by designing test fields that considered small‐field and tongue‐and‐groove effects. They determined the optimal DLG values by measuring them using an IC, and evaluated the GPR using diode array dosimeters. In contrast to their approach, this study presents a novel evaluation of the consistency of optimal DLG values across different diode array dosimeters, using IC‐based measurements as a reference.

This study has some limitations. First, although the DLG and LTF values interacted with the dose distribution and GPR, the DLG parameter was adjusted for a fixed LTF. Therefore, if LTF is adjusted, the optimal DLG values should be adjusted. For example, changing LTF from 1.5% to 1.9% shifted the optimal DLG values across all the evaluation criteria. Specifically, in patient “Prostate 1”, the optimal DLG changed from 0.12 to 0.10 cm for 3%/2 mm, from 0.12 to 0.10 cm for 2%/2 mm, from 0.12 to 0.10 cm for 1%/1 mm, and from 0.15 to 0.13 cm for dose difference analysis using ArcCHECK (Figure S1). These results indicate that LTF significantly affect the determination of the optimal DLG and should be carefully selected during beam model commissioning and validation. These findings are consistent with those reported by Isono et al.^14^ and Glenn et al.^22^ These studies highlighted the variability in DLG and LTF values used across different institutions. Second, this study included a limited number of plans (n = 5 per beam energy), which may have reduced the reliability of the statistical analysis. However, the cases were randomly selected to represent the typical treatment sites. Third, all data were acquired using a single LINAC model with high‐definition MLC and specific Eclipse parameters. Therefore, these findings may not be directly applicable to other devices or TPS configurations. Nevertheless, GPR can vary depending on the type of diode array dosimeter used. Thus, a GPR exceeding 95% under a single set of evaluation criteria does not necessarily guarantee that the selected DLG is optimal. This consideration is broadly applicable to commissioning other LINACs and TPS configurations. Fourth, because LTF and DLG vary with beam energy, focal spot size, MLC model, calculation algorithm, and field size,^14^, ^22^, ^23^, ^24^, ^25^ optimal DLG values may differ across institutions depending on their respective instruments and settings. Thus, individualized measurement of DLG is required.

## 5 CONCLUSION

We compared the optimal DLG values obtained using ArcCHECK, Delta4, SRS MapCHECK, and IC for 6 MV, 10 MV, 6 MV FFF, and 10 MV FFF. The average deviation was −0.35 ± 0.23 mm (range: −0.80 to 0.26 mm), resulting in approximately 1% dose discrepancies, which are clinically acceptable. These results suggest that optimal DLG values can be determined by systematically adjusting the DLG values within the Eclipse TPS and evaluating GPR and dose differences between recalculated doses and measurements obtained with diode array dosimeters—such as ArcCHECK, Delta4, and SRS MapCHECK—in the same way as with ICs. However, it should be noted that selecting a DLG value based solely on GPR exceeding 95% under limited and lenient conditions (e.g., 3%/2 mm) may be misleading. Stricter criteria, such as 2%/2 mm, 1%/1 mm, and dose difference evaluations are more useful for identifying clinically optimal DLG values.

## AUTHOR CONTRIBUTIONS

Yoshitsugu Matsumoto wrote the main script. Yoshitsugu Matsumoto and Ryosuke Sato analyzed data. All co‐authors have reviewed and edited to the work and have given approval to the submission.

## CONFLICT OF INTEREST STATEMENT

The authors have no relevant conflicts of interest to disclose.

## GENERATIVE AI AND LARGE LANGUAGE MODELS

AI‐based language assistance ChatGPT was used to improve the clarity and fluency of English expressions in this manuscript. The authors take full responsibility for the content.

## Supporting information



Supporting Information

## Data Availability

The data are stored at the author's institution and are available upon request.
