# Supplementary material for: Optimizing dosimetric leaf gap parameters for radiation therapy: A comparative study using three diode‐array verification tools and an ionization chamber
Source: J Appl Clin Med Phys. 2025 Oct 28;26(11):e70280. doi: 10.1002/acm2.70280 (PMC12560010; doi:10.1002/acm2.70280)
Supplement: Supplementary file 1 — Supporting Information [file ACM2-26-e70280-s001.docx]

**Supplementary Figure S1.**
This figure illustrates how changes in the leaf transmission factor (LTF) affect the relationship between optimal dosimetric leaf gap (DLG), gamma pass rate (GPR), and dose difference (DD) for ArcCHECK. Data are shown for the 10 MV VMAT “Prostate 1” plan under four evaluation conditions: 3%/2 mm and 2%/2 mm global GPR, 1%/1 mm local GPR, and DD. The x-axis represents the change in DLG values, while the y-axis shows the corresponding change in GPR or DD. The results demonstrate that modifying the LTF value (1.5% vs. 1.9%) causes a shift in both the point of highest GPR and the point of minimum DD, indicating that the optimal DLG value varies depending on the LTF setting.
